# COVID-19 and long-term risk of incident laboratory-defined micronutrient deficiency in patients with type 2 diabetes: a multicenter propensity score–matched cohort study

**DOI:** 10.3389/fnut.2026.1915257

**Published:** 2026-08-31

**Authors:** I-Wen Chen, Li-Chen Chang, Kuo-Chuan Hung

**Affiliations:** 1 Department of Anesthesiology, Chi Mei Hospital, Liouying, Tainan City, Taiwan; 2 Department of Anesthesiology, E-Da Hospital, I-Shou University, Kaohsiung City, Taiwan; 3 Department of Anesthesiology, Chi Mei Medical Center, Tainan City, Taiwan

**Keywords:** COVID-19, magnesium deficiency, SARS-CoV-2, type 2 diabetes mellitus, vitamin B12 deficiency, vitamin D deficiency

## Abstract

**Background:**

COVID-19 has been linked to broad post-acute sequelae, but delayed micronutrient deficiency remains insufficiently characterized. Whether COVID-19 increases long-term risks of laboratory-defined vitamin D, magnesium, and vitamin B12 deficiencies among patients with type 2 diabetes mellitus is unclear.

**Methods:**

Using the predominantly US-based TriNetX network, we identified adults with type 2 diabetes mellitus (T2DM) during 2020–2022 who had COVID-19 and matched controls without COVID-19. A 12-month landmark design excluded pre-existing deficiencies, skeletal outcomes, and early deaths. After 1:1 propensity score matching, Cox models estimated hazard ratios (HRs) for incident deficiencies and skeletal outcomes over an up-to-5-year analytic window, with tested-cohort, severity-gradient, competing-risk, and E-value analyses.

**Results:**

After matching, 645,168 patients remained per group. COVID-19 was associated with higher risks of vitamin D deficiency (HR, 1.79), magnesium deficiency (HR, 1.77), vitamin B12 deficiency (HR, 1.56), osteoporosis (HR, 1.55), osteoporotic fracture (HR, 1.73), femur fracture (HR, 1.58), and death (HR, 1.59; all *p* < 0.001). Among patients with corresponding nutrient testing, risks remained higher for vitamin D deficiency (19.1% vs. 12.8%, HR, 1.68), magnesium deficiency (25.1% vs. 20.0%, HR, 1.47), and vitamin B12 deficiency (4.4% vs. 3.4%, HR, 1.42, all *p* < 0.001). In tested cohorts, cumulative-incidence estimates remained higher after COVID-19 despite greater competing mortality. Risks were generally higher after severe infection.

**Conclusion:**

In patients with T2DM, COVID-19 was associated with increased long-term risks of laboratory-defined micronutrient deficiencies and skeletal outcomes. These hypothesis-generating findings warrant prospective confirmation.

## 1 Introduction

The coronavirus disease 2019 (COVID-19) pandemic has affected hundreds of millions of individuals worldwide, and attention has increasingly shifted from acute infection to its long-term consequences (1–3). Accumulating evidence indicates that survivors of severe acute respiratory syndrome coronavirus 2 (SARS-CoV-2) infection remain at increased risk for a broad range of newly incident multisystem conditions during the post-acute phase, collectively described as post-acute sequelae of SARS-CoV-2 infection (4, 5). Given the large cumulative number of infected individuals, even modest increases in delayed chronic conditions may impose a substantial population-level burden (6, 7), underscoring the need to systematically characterize long-term sequelae.

Nutritional status is closely linked to infection, inflammation, immune regulation, and chronic metabolic disease (8, 9). However, the relationship between COVID-19 and micronutrient status has largely been studied in one direction. Most prior investigations have evaluated nutrient deficiency, particularly vitamin D deficiency, as a baseline predictor of SARS-CoV-2 susceptibility, COVID-19 hospitalization, disease severity, or mortality, rather than as a potential post-acute consequence of infection (10–13). Whether SARS-CoV-2 infection itself is associated with an increased subsequent risk of incident micronutrient deficiency remains comparatively underexplored. Such an association is biologically plausible, as COVID-19 may contribute to sustained systemic inflammation, reduced dietary intake, decreased sunlight exposure, gastrointestinal dysfunction affecting vitamin B12 absorption, and renal or metabolic disturbances that alter magnesium and vitamin D homeostasis (14–18).

Nutrition has also emerged as an important aspect of post-COVID management. Recent scoping and systematic reviews have highlighted the potential roles of diet and micronutrients, including vitamin D, B-group vitamins, zinc, and magnesium, in post-acute sequelae, and some experts have advocated routine nutritional assessment during post-COVID follow-up (19–21). However, existing studies have primarily focused on nutritional interventions and recovery-related outcomes, such as supplementation, malnutrition, sarcopenia, and body composition, with relatively short follow-up periods. Consequently, whether prior COVID-19 is associated with delayed-onset deficiencies across multiple micronutrients, and whether such associations persist for several years after acute infection, remains unclear.

This study sought to assess whether, among patients with type 2 diabetes mellitus who survived acute infection, COVID-19 is associated with a higher long-term risk of incident vitamin D, magnesium, and vitamin B12 deficiency. We also explored whether these associations varied according to COVID-19 severity and examined related skeletal outcomes to provide additional clinical context.

## 2 Methods

### 2.1 Study design and data source

We conducted this retrospective, multicenter cohort study using the TriNetX Global Collaborative Network, a federated electronic health record research platform that provides de-identified, patient-level data from participating healthcare organizations. The database includes longitudinal information on demographics, diagnoses, procedures, medications, laboratory measurements, healthcare encounters, and vital status. The TriNetX platform has been widely used in published clinical research across diverse medical specialties (22–25). The analysis was performed within the TriNetX Analytics platform using a prespecified Compare Outcomes framework. Data were extracted from the TriNetX Global Collaborative Network on June 9, 2026. The study was approved by the Institutional Review Board of Chi Mei Medical Center, and the requirement for informed consent was waived because only de-identified data were used. All procedures were conducted in accordance with the Declaration of Helsinki.

### 2.2 Study population and exposure definition

We identified adult patients aged 18 years or older with type 2 diabetes mellitus between January 1, 2020 and June 30, 2022. Type 2 diabetes mellitus was defined using ICD-10-CM code E11. The exposed cohort included patients with type 2 diabetes mellitus who had evidence of COVID-19 during the study period, defined by either ICD-10-CM code U07.1 or a recorded positive SARS-CoV-2 RNA test. The first qualifying COVID-19 episode defined the exposure, and subsequent reinfections were not modeled as time-varying exposures. The comparison cohort included patients with type 2 diabetes mellitus who had no recorded COVID-19 diagnosis and no recorded positive SARS-CoV-2 RNA test during the same period. Baseline covariates were assessed over the 5 years preceding the index date, during which patients were required to have at least one documented healthcare encounter to ensure adequate baseline clinical information.

For the exposed cohort, the index date was defined as the first recorded COVID-19 diagnosis or positive SARS-CoV-2 RNA test during the eligibility period. For the comparison cohort, the index date was defined as the first healthcare encounter during the eligibility period at which type 2 diabetes mellitus (T2DM) was documented, in the absence of any recorded COVID-19 diagnosis or positive SARS-CoV-2 RNA test. To assemble a cohort at risk for incident nutritional deficiency and related bone outcomes, we excluded patients with any evidence of vitamin D deficiency, magnesium deficiency, vitamin B12 deficiency, osteoporosis, or osteoporotic fracture before or within 12 months after the index date (landmark analysis). Vitamin D deficiency was defined as serum 25-hydroxyvitamin D < 20 ng/mL (26); magnesium deficiency as serum magnesium <1.70 mg/dL (27); and vitamin B12 deficiency as serum vitamin B12 ≤ 200 pg./mL (28). We also excluded patients who, before the index date, had type 1 diabetes mellitus, gestational diabetes mellitus, stage 4 or 5 chronic kidney disease, ESRD, dialysis dependence, or a history of bariatric surgery. Patients who died within the first 12 months after the index date were not included in the landmark cohort. The complete list of variables used for exclusion criteria is provided in Supplementary Table 1.

### 2.3 Landmark design and follow-up

The same 12-month landmark design was applied to both cohorts to reduce reverse causation, pre-existing but previously undocumented nutritional deficiency, and short-term ascertainment related to acute COVID-19 infection. Only patients who remained alive and free of the prespecified nutritional deficiency and bone outcomes throughout the first 12 months after the index date were eligible for outcome assessment. The primary follow-up window spanned days 365 to 1825 after the index date. Outcomes occurring during this late follow-up window were considered incident events. Because cohort entry occurred between January 1, 2020, and June 30, 2022, and data were extracted on June 9, 2026, the maximum potential observation period from the index date ranged from approximately 47 to 77 months. Accordingly, the reported 5-year estimates represent an up-to-5-year analytic window in which later-indexed patients contributed less post-landmark person-time and the later portion of follow-up was informed disproportionately by patients indexed earlier in the pandemic.

### 2.4 Propensity score matching

To reduce baseline differences between the COVID-19 and control cohorts, 1:1 propensity score matching was performed using a greedy nearest-neighbor algorithm with a caliper of 0.1 pooled standard deviations of the logit of the propensity score. Covariates entered into the propensity score model comprised demographic characteristics, comorbidities, diabetes-related complications, medications, vaccination status assessed before the index date, and laboratory-based markers of baseline health status. The complete list of variables used for propensity score matching is provided in Supplementary Table 2. Covariate balance was assessed using standardized mean differences, with values <0.10 considered to indicate acceptable balance. Propensity score density plots were used to visually evaluate the overlap between cohorts before and after matching.

### 2.5 Outcomes

The primary outcome was incident vitamin D deficiency during follow-up, defined as serum 25-hydroxyvitamin D < 20 ng/mL. Secondary outcomes included magnesium deficiency, vitamin B12 deficiency, osteoporosis composite, osteoporotic fracture, and femur fracture. Supportive outcomes included all-cause mortality, any recorded healthcare encounter without classification by encounter modality, and vitamin D measurement during follow-up to provide information on competing risk, healthcare utilization, and laboratory surveillance. The primary analysis used a 12-month landmark design and assessed outcomes from 365 to 1825 days after the index date (an up-to-5-year analytic window). A separate 3-year analysis was also performed to evaluate whether the magnitude of the association differed over a shorter analytic window. For each endpoint, patients were followed independently until the first occurrence of that endpoint, death, the last available electronic health record encounter, or the end of the study period, whichever occurred first. The occurrence of one nutritional deficiency did not remove a patient from analyses of the other endpoints.

Magnesium deficiency was defined as serum magnesium <1.70 mg/dL, and vitamin B12 deficiency was defined as serum vitamin B12 ≤ 200 pg./mL. The osteoporosis composite included osteoporosis with current pathological fracture (ICD-10-CM code M80) and osteoporosis without current pathological fracture (ICD-10-CM code M81). Osteoporotic fracture was defined as osteoporosis with current pathological fracture (ICD-10-CM code M80), and femur fracture was defined using ICD-10-CM code S72. Mortality was identified using recorded deceased status or ICD-10-CM code R99.

### 2.6 Analysis among patients with nutrient testing during follow-up

Because nutritional deficiencies can only be detected when laboratory testing is performed, an additional analysis was restricted to patients who had at least one corresponding nutrient measurement during follow-up. This restriction conditions on post-baseline testing, which may be influenced by both COVID-19 exposure and clinical manifestations related to underlying nutritional status and may therefore introduce selection bias rather than eliminate ascertainment bias. Separate tested cohorts were created for vitamin D, magnesium, and vitamin B12 analyses. Within each tested cohort, incident deficiency was defined by the first laboratory-defined deficiency during the follow-up window. Absolute risk differences were calculated as the event proportion in the COVID-19 group minus that in the control group, and these tested-cohort analyses were interpreted as triangulating evidence rather than as bias-free estimates.

### 2.7 Sensitivity and subgroup analyses

Several sensitivity analyses were conducted to evaluate the robustness of the primary findings and address potential sources of bias. Model I was restricted to patients who survived through follow-up, minimizing the influence of differential mortality. Model II was confined to patients with at least one healthcare visit during follow-up, reducing bias related to unequal healthcare utilization and outcome ascertainment. Model III restricted the study period to the later pandemic period from June 2021 to June 2022 while retaining the 12-month landmark design to account for temporal changes in SARS-CoV-2 variants, vaccination coverage, and clinical management. Prespecified subgroup analyses were performed according to sex and age group, with 1:1 propensity score matching repeated separately within each sex- and age-specific stratum. Age was categorized as 18–65 years and >65 years. Hazard ratios were estimated within each subgroup, and interaction testing was performed to evaluate whether the association between COVID-19 and each outcome differed across strata.

### 2.8 Exposure-gradient analysis

To explore whether greater COVID-19 severity was associated with a stronger risk pattern, an exposure-gradient analysis compared patients with severe COVID-19 with matched controls. Severe COVID-19 was defined as COVID-19 with hospitalization or intensive care unit admission recorded on the index date. The same outcome definitions and statistical approach used in the primary analysis were applied to the severe COVID-19 analysis.

### 2.9 Descriptive competing-risk analysis

Because nutritional deficiencies can only be identified when laboratory testing is performed, the competing-risk analysis was conducted among patients with nutrient testing during follow-up. Within these tested cohorts, death may preclude subsequent diagnosis of nutritional deficiency; therefore, a descriptive competing-risk analysis was performed using the Aalen–Johansen method, with death treated as a competing event. This analysis was considered supportive and descriptive rather than the primary inferential approach.

### 2.10 Statistical analysis

All primary analyses were conducted on the propensity score–matched cohorts. Continuous variables were summarized as means with standard deviations, and categorical variables as counts with percentages; between-group comparisons of baseline characteristics were described using standardized mean differences rather than significance testing, because conventional *p-*values are sensitive to the very large sample size. Time-to-event outcomes were analyzed using Cox proportional hazards regression, with the index date as the time origin and follow-up beginning at the start of the post-landmark window. For each outcome, the association between COVID-19 and the risk of the event was expressed as a hazard ratio (HR) with its corresponding 95% confidence interval (CI), with the control cohort serving as the reference group. The proportional hazards assumption was evaluated for the primary outcome within the TriNetX platform. When the diagnostic indicated nonproportional hazards, Cox estimates were interpreted as average hazard ratios over the specified analytic window rather than as constant effects throughout follow-up. Accordingly, the existing up-to-3-year and up-to-5-year estimates were reported together to characterize differences in the magnitude of association across analytic windows. E-values were calculated for the primary outcome, secondary clinical outcomes, all-cause mortality, and tested-cohort nutrient outcomes to assess the minimum strength of association that an unmeasured confounder would need to have with both exposure and outcome, beyond the measured covariates, to fully explain the observed association. E-values quantify robustness to unmeasured confounding but do not address differential outcome ascertainment or selection bias. Missing data were not imputed; patients without recorded values for laboratory covariates were retained using a missing-indicator approach within the TriNetX propensity score model. Analyses were conducted using available data within the TriNetX platform. Two-sided *p-*values were reported, with *p* < 0.05 considered statistically significant for the primary outcome. Secondary outcomes, sensitivity analyses, subgroup analyses, exposure-gradient analyses, tested-cohort analyses, and competing-risk analyses were considered exploratory, and no adjustment for multiple comparisons was performed.

## 3 Results

### 3.1 Patient selection and baseline characteristics

**Figure 1:**
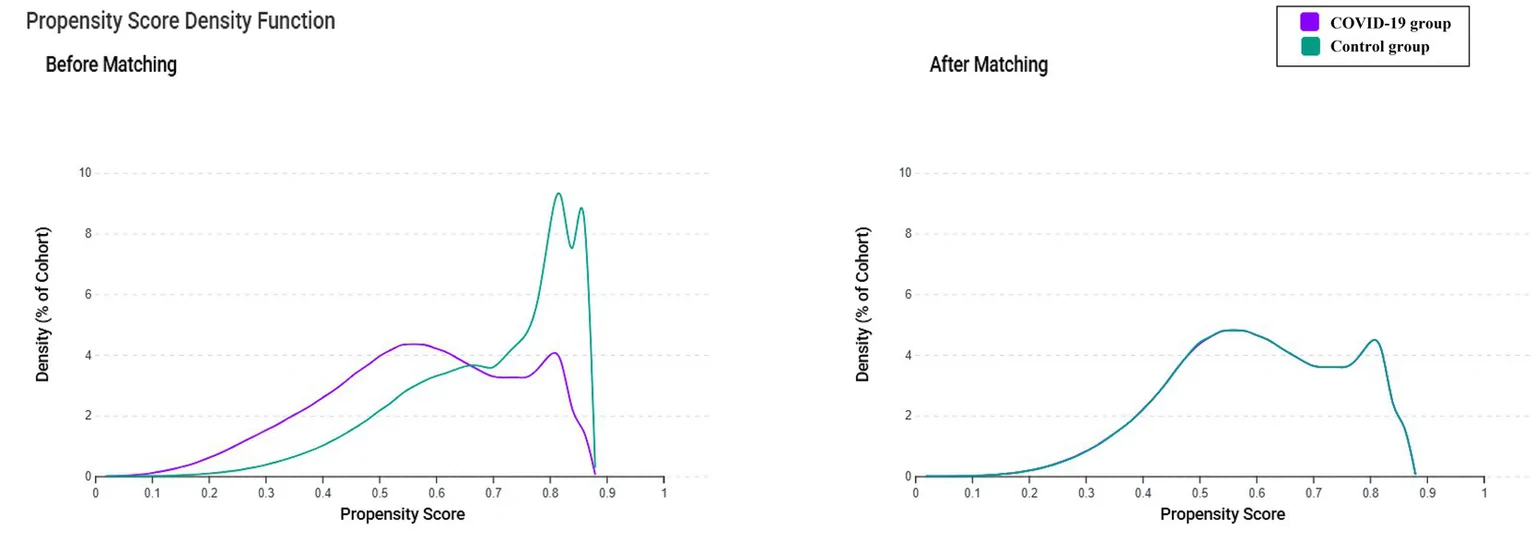
Propensity score distributions before and after matching. Propensity score density plots comparing patients with type 2 diabetes with COVID-19 and matched controls before and after 1:1 propensity score matching. Before matching, the two cohorts showed partial separation in propensity score distributions. After matching, the distributions showed substantial overlap, indicating improved covariate balance between groups.

After the study eligibility criteria and 12-month landmark design were applied, the pre-matching cohort comprised 713,415 patients with type 2 diabetes and COVID-19 and 1,399,912 patients with type 2 diabetes without COVID-19 (Table 1). Compared with controls, patients in the COVID-19 group had higher baseline burdens of obesity, hypertension, dyslipidemia, ischemic heart disease, chronic kidney disease, heart failure, chronic obstructive pulmonary disease, and diabetes-related complications before matching. After 1:1 propensity score matching, 645,168 patients remained in each cohort. Baseline characteristics were well balanced after matching, with all standardized mean differences <0.10. Mean age was similar between groups (62.0 years in the COVID-19 group vs. 62.1 years in controls), as was the proportion of women (46.0% vs. 45.3%). After matching, propensity score density plots demonstrated improved overlap between cohorts, consistent with adequate covariate balance (Table 1 and Figure 1).

**Table 1:** Baseline characteristics of patients with type 2 diabetes with and without COVID-19 before and after propensity score matching.

Variables	Before matching		After matching			
	COVID-19 group (*n* = 713,415)	Control group (*n* = 1,399,912)	SMD	COVID-19 group (*n* = 645,168)	Control group (*n* = 645,168)	SMD
Patient characteristics						
Age at index (years)	62.2 ± 14.1	62.4 ± 13.6	0.011	62.0 ± 14.2	62.1 ± 13.6	0.001
Female	327,030 (45.8)	642,410 (45.9)	0.001	296,652 (46.0)	292,139 (45.3)	0.014
BMI > 30 kg/m^2^	334,885 (46.9)	419,746 (30.0)	0.354	285,439 (44.2)	293,862 (45.5)	0.026
White	427,082 (59.9)	688,523 (49.2)	0.216	377,164 (58.5)	384,925 (59.7)	0.024
Black or African American	124,733 (17.5)	199,258 (14.2)	0.089	110,826 (17.2)	113,257 (17.6)	0.010
Asian	38,064 (5.3)	97,219 (6.9)	0.067	36,587 (5.7)	39,885 (6.2)	0.022
Comorbidities and health services						
Essential (primary) hypertension	440,682 (61.8)	587,240 (41.9)	0.405	378,966 (58.7)	386,198 (59.9)	0.023
Disorders of lipoprotein metabolism and other lipidemias	386,928 (54.2)	529,450 (37.8)	0.334	331,956 (51.5)	336,990 (52.2)	0.016
Overweight and obesity	220,976 (31.0)	235,449 (16.8)	0.337	179,013 (27.7)	178,948 (27.7)	0.000
Neoplasms	183,302 (25.7)	223,571 (16.0)	0.241	150,695 (23.4)	152,143 (23.6)	0.005
Osteoarthritis	165,244 (23.2)	180,977 (12.9)	0.269	131,562 (20.4)	130,613 (20.2)	0.004
Ischemic heart diseases	147,622 (20.7)	167,872 (12.0)	0.237	116,081 (18.0)	115,457 (17.9)	0.003
Nicotine dependence	89,564 (12.6)	87,892 (6.3)	0.216	69,690 (10.8)	68,859 (10.7)	0.004
Diseases of liver	72,897 (10.2)	81,378 (5.8)	0.163	57,700 (8.9)	56,371 (8.7)	0.007
Chronic kidney disease	73,950 (10.4)	79,632 (5.7)	0.173	57,042 (8.8)	56,521 (8.8)	0.003
T2DM with neurological complications	69,327 (9.7)	71,555 (5.1)	0.176	53,145 (8.2)	52,634 (8.2)	0.003
Cerebrovascular diseases	68,302 (9.6)	77,127 (5.5)	0.154	53,091 (8.2)	52,572 (8.1)	0.003
T2DM with kidney complications	65,365 (9.2)	74,715 (5.3)	0.148	50,864 (7.9)	50,561 (7.8)	0.002
Heart failure	65,670 (9.2)	53,831 (3.8)	0.218	45,081 (7.0)	43,540 (6.7)	0.009
Other chronic obstructive pulmonary disease	60,384 (8.5)	52,222 (3.7)	0.199	42,480 (6.6)	40,992 (6.4)	0.009
T2DM with ophthalmic complications	29,217 (4.1)	39,971 (2.9)	0.068	23,894 (3.7)	24,281 (3.8)	0.003
T2DM with circulatory complications	32,086 (4.5)	30,399 (2.2)	0.130	23,664 (3.7)	23,295 (3.6)	0.003
Disorders of bone density and structure	27,606 (3.9)	29,670 (2.1)	0.103	21,741 (3.4)	21,493 (3.3)	0.002
Alcohol related disorders	20,621 (2.9)	17,660 (1.3)	0.114	14,965 (2.3)	14,475 (2.2)	0.005
Malnutrition	6,576 (0.9)	3,399 (0.2)	0.089	3,432 (0.5)	3,112 (0.5)	0.007
Medications						
Antilipemic agents	337,999 (47.4)	440,399 (31.5)	0.330	286,257 (44.4)	290,231 (45.0)	0.012
Metformin	276,901 (38.8)	386,398 (27.6)	0.240	240,899 (37.3)	247,679 (38.4)	0.022
Insulins and analogues	193,149 (27.1)	201,844 (14.4)	0.316	154,413 (23.9)	153,339 (23.8)	0.004
SARS-cov-2 (COVID-19) vaccine	76,310 (10.7)	64,590 (4.6)	0.230	48,915 (7.6)	43,457 (6.7)	0.033
SGLT2 inhibitors	59,615 (8.4)	71,295 (5.1)	0.131	48,643 (7.5)	48,714 (7.6)	0.000
GLP-1 analogues	61,048 (8.6)	64,384 (4.6)	0.160	48,515 (7.5)	47,893 (7.4)	0.004
Laboratory data						
eGFR≥60 mL/min/1.73 m^2^	490,316 (68.7)	645,133 (46.1)	0.470	427,595 (66.3)	430,804 (66.8)	0.011
Hemoglobin ≥12 g/dL	476,655 (66.8)	561,480 (40.1)	0.556	410,499 (63.6)	412,322 (63.9)	0.006
Albumin ≥3.5 g/dL	441,169 (61.8)	511,139 (36.5)	0.524	377,053 (58.4)	376,550 (58.4)	0.002
HbA1c ≥ 9%	104,472 (14.6)	137,748 (9.8)	0.147	89,715 (13.9)	89,661 (13.9)	0.000

Data are presented as number (%) or mean ± standard deviation. Standardized mean differences <0.10 were considered to indicate acceptable covariate balance. BMI, body mass index; COVID-19, coronavirus disease 2019; eGFR, estimated glomerular filtration rate; GLP-1, glucagon-like peptide-1; HbA1c, hemoglobin A1c; SGLT2, sodium-glucose cotransporter 2; SMD, standardized mean difference; T2DM, type 2 diabetes mellitus.

### 3.2 Primary and secondary outcomes

The mean follow-up duration was 1,264.6 days (SD, 601.7) in the COVID-19 cohort and 1,378.2 days (SD, 583.9) in the control cohort. The corresponding median follow-up durations were 1,495 and 1,655 days, respectively. Over the up-to-5-year analytic window, COVID-19 was associated with a higher risk of incident vitamin D deficiency (HR, 1.79; 95% CI, 1.75–1.84) (Table 2). These whole-cohort estimates for laboratory-defined nutrient deficiencies were testing-inclusive, reflecting both the probability of undergoing nutrient measurement and the probability of meeting the deficiency threshold conditional on measurement. For this primary outcome, the proportional-hazards diagnostic indicated evidence of nonproportional hazards (*p* < 0.001); therefore, the HR should be interpreted as an average association over the up-to-5-year analytic window rather than as a constant relative hazard throughout follow-up. Increased risks were also observed for osteoporosis composite (HR, 1.55; 95% CI, 1.51–1.58), osteoporotic fracture (HR, 1.73; 95% CI, 1.63–1.85), femur fracture (HR, 1.58; 95% CI, 1.52–1.64), vitamin B12 deficiency (HR, 1.56; 95% CI, 1.50–1.63), and magnesium deficiency (HR, 1.77; 95% CI, 1.75–1.80). Mortality was also higher in the COVID-19 group (HR, 1.59; 95% CI, 1.56–1.61), whereas any healthcare visit was slightly less frequent (HR, 0.97; 95% CI, 0.96–0.97). Vitamin D measurement was modestly more common after COVID-19 (HR, 1.21; 95% CI, 1.20–1.22) (Table 2). For the primary outcome of vitamin D deficiency, the E-value was 2.98 for the point estimate and 2.90 for the lower confidence interval bound, indicating that an unmeasured confounder would need to be associated with both COVID-19 and vitamin D deficiency by a risk ratio of at least 2.98 each, above and beyond the measured covariates, to fully explain away the observed association.

**Table 2:** Association between COVID-19 and up-to-5-year risks of incident nutritional deficiencies and related outcomes.

Outcome	COVID-19 group (*n* = 645,168)	Control group (*n* = 645,168)	HR (95% CI)	*p-*value	*E*-value, point/lower CI
	Events (%)	Events (%)			
Primary outcome					
Vitamin D deficiency	16,772 (2.60)	10,695 (1.66)	1.79 (1.75–1.84)	<0.001	2.98/2.90
Secondary outcomes					
Osteoporosis composite	17,384 (2.69)	12,906 (2.00)	1.55 (1.51–1.58)	<0.001	2.47/2.39
Osteoporotic fracture	2,363 (0.37)	1,577 (0.24)	1.73 (1.63–1.85)	<0.001	2.85/2.64
Femur fracture	6,045 (0.94)	4,319 (0.67)	1.58 (1.52–1.64)	<0.001	2.54/2.41
Vitamin B12 deficiency	5,257 (0.82)	3,844 (0.60)	1.56 (1.50–1.63)	<0.001	2.49/2.37
Magnesium deficiency	37,344 (5.79)	24,468 (3.79)	1.77 (1.75–1.80)	<0.001	2.94/2.90
Mortality and healthcare-utilization marker					
Any recorded healthcare encounter	553,197 (85.75)	569,588 (88.29)	0.97 (0.96–0.97)	<0.001	—
Vitamin D measurement	85,853 (13.31)	79,237 (12.28)	1.21 (1.20–1.22)	<0.001	—
Mortality	33,993 (5.27)	24,336 (3.77)	1.59 (1.56–1.61)	<0.001	2.56/2.49

Data are presented as number of events (%). Hazard ratios were estimated using Cox proportional hazards models. Any recorded healthcare encounter was assessed as a general healthcare-utilization marker. CI, confidence interval; COVID-19, coronavirus disease 2019; HR, hazard ratio.

### 3.3 Primary and secondary outcomes at up-to-3-year follow-up

In the up-to-3-year analysis, the risk pattern was directionally consistent with the 5-year findings (Table 3). Vitamin D deficiency occurred in 9,624 patients (1.49%) in the COVID-19 group and 4,023 patients (0.62%) in the control group (HR, 2.55; 95% CI, 2.46–2.64; *p* < 0.001). COVID-19 was also associated with higher risks of osteoporosis composite (HR, 2.16; 95% CI, 2.08–2.23), osteoporotic fracture (HR, 2.45; 95% CI, 2.20–2.73), femur fracture (HR, 1.88; 95% CI, 1.78–1.98), vitamin B12 deficiency (HR, 2.24; 95% CI, 2.10–2.39), and magnesium deficiency (HR, 2.66; 95% CI, 2.59–2.73) (all *p* < 0.001). Mortality was higher in the COVID-19 group (2.96% vs. 1.53%, HR, 2.04, 95% CI, 1.99–2.09), while any healthcare visit remained slightly lower (83.04% vs. 85.75%, HR, 0.96, 95% CI, 0.96–0.97).

**Table 3:** Association between COVID-19 and up-to-3-year risks of incident nutritional deficiencies and related outcomes.

Outcome	COVID-19 group (*n* = 645,168)	Control group (*n* = 645,168)	HR (95% CI)	*p-*value
	Events (%)	Events (%)		
Vitamin D deficiency	9,624 (1.49)	4,023 (0.62)	2.55 (2.46–2.64)	<0.001
Osteoporosis composite	9,420 (1.46)	4,653 (0.72)	2.16 (2.08–2.23)	<0.001
Osteoporotic fracture	1,115 (0.17)	483 (0.08)	2.45 (2.20–2.73)	<0.001
Femur fracture	3,598 (0.56)	2,023 (0.31)	1.88 (1.78–1.98)	<0.001
Vitamin B12 deficiency	2,860 (0.44)	1,357 (0.21)	2.24 (2.10–2.39)	<0.001
Magnesium deficiency	20,883 (3.24)	8,430 (1.31)	2.66 (2.59–2.73)	<0.001
Any recorded healthcare encounter	535,717 (83.04)	553,245 (85.75)	0.96 (0.96–0.97)	<0.001
Vitamin D measurement	59,983 (9.30)	52,416 (8.12)	1.21 (1.20–1.23)	<0.001
Mortality	19,073 (2.96)	9,894 (1.53)	2.04 (1.99–2.09)	<0.001

Data are presented as number of events (%). Hazard ratios were estimated using Cox proportional hazards models. Any recorded healthcare encounter was assessed as a general healthcare-utilization marker. CI, confidence interval; COVID-19, coronavirus disease 2019; HR, hazard ratio.

### 3.4 Sensitivity and subgroup analyses

All subsequent sensitivity, subgroup, tested-cohort, exposure-gradient, and competing-risk analyses were considered exploratory. The associations remained robust across sensitivity analyses (Table 4). In patients who survived during follow-up, COVID-19 remained associated with vitamin D deficiency (HR, 1.78), osteoporosis composite (HR, 1.54), vitamin B12 deficiency (HR, 1.57), and magnesium deficiency (HR, 1.78). Similar results were observed among patients with at least one healthcare visit during follow-up. In the later-pandemic analysis restricted to June 2021–June 2022, the associations were attenuated but remained statistically significant for all outcomes, including vitamin D deficiency (HR, 1.38), vitamin B12 deficiency (HR, 1.23), and magnesium deficiency (HR, 1.45).

**Table 4:** Sensitivity analyses for the association between COVID-19 and risks of nutritional deficiencies and related outcomes.

Outcomes	Model I (*n* = 612,466)		Model II (*n* = 514,830)		Model III (*n* = 387,962)	
	HR (95% CI)	*p-*value	HR (95% CI)	*p-*value	HR (95% CI)	*p-*value
Vitamin D deficiency	1.78 (1.73–1.82)	<0.001	1.78 (1.74–1.83)	<0.001	1.38 (1.34–1.42)	<0.001
Osteoporosis composite	1.54 (1.50–1.58)	<0.001	1.55 (1.51–1.58)	<0.001	1.26 (1.23–1.29)	<0.001
Osteoporotic fracture	1.70 (1.59–1.82)	<0.001	1.79 (1.68–1.92)	<0.001	1.42 (1.32–1.52)	<0.001
Femur fracture	1.57 (1.50–1.64)	<0.001	1.64 (1.57–1.70)	<0.001	1.36 (1.30–1.43)	<0.001
Vitamin B12 deficiency	1.57 (1.50–1.64)	<0.001	1.57 (1.51–1.64)	<0.001	1.23 (1.17–1.29)	<0.001
Magnesium deficiency	1.78 (1.75–1.82)	<0.001	1.76 (1.74–1.80)	<0.001	1.45 (1.42–1.47)	<0.001

Model I included only patients who survived during follow-up. Model II included only patients with at least one healthcare visit during follow-up. Model III restricted the study period to June 2021 through June 2022 while retaining the 12-month landmark design. Hazard ratios were estimated using Cox proportional hazards models. CI, confidence interval; COVID-19, coronavirus disease 2019; HR, hazard ratio.

In subgroup analyses, the association between COVID-19 and nutritional deficiency outcomes was generally consistent across sex and age groups (Tables 5, 6). The risk of vitamin D deficiency was similar between sexes (Table 5), with hazard ratios of 1.80 in men and 1.75 in women and no significant interaction (*p* for interaction = 0.248). Significant sex interactions were observed for osteoporosis composite and magnesium deficiency, with stronger relative associations in men. By age (Table 6), the association with vitamin D deficiency was stronger among patients >65 years than among those aged 18–65 years (HR, 1.87 vs. 1.70; *p* for interaction<0.001). Other outcomes showed no major age-related interaction.

**Table 5:** Subgroup analyses by sex for the association between COVID-19 and risks of nutritional deficiencies and related outcomes.

Outcomes	Male (*n* = 350,898 for each group)		Female (*n* = 293,137 for each group)		*p* for interaction
	HR (95% CI)	*p-*value	HR (95% CI)	*p-*value	
Vitamin D deficiency	1.80 (1.74–1.86)	<0.001	1.75 (1.69–1.81)	<0.001	0.248
Osteoporosis composite	1.71 (1.62–1.81)	<0.001	1.53 (1.49–1.56)	<0.001	<0.001
Osteoporotic fracture	1.78 (1.58–1.99)	<0.001	1.73 (1.60–1.87)	<0.001	0.690
Femur fracture	1.63 (1.53–1.72)	<0.001	1.57 (1.49–1.66)	<0.001	0.356
Vitamin B12 deficiency	1.57 (1.48–1.66)	<0.001	1.56 (1.47–1.66)	<0.001	0.881
Magnesium deficiency	1.83 (1.79–1.87)	<0.001	1.70 (1.66–1.74)	<0.001	<0.001

Data are presented as hazard ratios with 95% confidence intervals and *p*-values. Hazard ratios were estimated separately in male and female patients after 1:1 propensity score matching performed within each sex stratum, such that each subgroup analysis was based on an independently matched cohort. *p*-values for interaction were used to assess whether the association between COVID-19 and each outcome differed by sex. CI, confidence interval; COVID-19, coronavirus disease 2019; HR, hazard ratio.

**Table 6:** Subgroup analyses by age for the association between COVID-19 and risks of nutritional deficiencies and related outcomes.

Outcomes	18–65 years (*n* = 269,806 for each group)		>65 years (*n* = 348,472 for each group)		*p* for interaction
	HR (95% CI)	*p-*value	HR (95% CI)	*p-*value	
Vitamin D deficiency	1.70 (1.64–1.75)	<0.001	1.87 (1.80–1.94)	<0.001	<0.001
Osteoporosis composite	1.69 (1.59–1.79)	<0.001	1.61 (1.57–1.65)	<0.001	0.145
Osteoporotic fracture	1.78 (1.47–2.15)	<0.001	1.76 (1.64–1.89)	<0.001	0.914
Femur fracture	1.61 (1.45–1.78)	<0.001	1.53 (1.46–1.60)	<0.001	0.382
Vitamin B12 deficiency	1.57 (1.46–1.69)	<0.001	1.57 (1.49–1.65)	<0.001	1.000
Magnesium deficiency	1.79 (1.74–1.84)	<0.001	1.76 (1.73–1.80)	<0.001	0.335

Data are presented as hazard ratios with 95% confidence intervals and *p*-values. Hazard ratios were estimated separately among patients aged 18–65 years and >65 years after separate 1:1 propensity score matching within each age stratum, such that each subgroup analysis was based on an independently matched cohort. *p*-values for interaction were used to assess whether the association between COVID-19 and each outcome differed by age group. CI, confidence interval; COVID-19, coronavirus disease 2019; HR, hazard ratio.

### 3.5 Risk of incident nutritional deficiencies among patients with relevant nutrient testing during follow-up

Among patients with corresponding nutrient testing during follow-up, COVID-19 remained associated with higher risks of laboratory-defined deficiencies (Table 7). Vitamin D deficiency occurred in 15,748 of 82,290 tested patients (19.1%) in the COVID-19 group and 10,513 of 82,290 tested controls (12.8%), corresponding to an absolute risk difference of 6.4% and an HR of 1.68 (95% CI, 1.64–1.72, *p* < 0.001). Magnesium deficiency occurred in 25.1% versus 20.0% of tested patients (absolute risk difference, 5.1%; HR, 1.47; 95% CI, 1.44–1.49), and vitamin B12 deficiency occurred in 4.4% versus 3.4% (absolute risk difference, 1.0%; HR, 1.42; 95% CI, 1.36–1.48).

**Table 7:** Risk of incident nutritional deficiencies among patients with relevant nutrient testing during follow-up.

Nutritional deficiency	Matched tested cohort, *n* per group	COVID-19 group, events *n* (%)	Control group, events *n* (%)	Absolute risk difference, %	HR (95% CI)	*p-*value	*E*-value, point/lower CI
Vitamin D deficiency	82,290	15,748 (19.1)	10,513 (12.8)	6.4	1.68 (1.64–1.72)	<0.001	2.75/2.66
Magnesium deficiency	136,522	34,219 (25.1)	27,293 (20.0)	5.1	1.47 (1.44–1.49)	<0.001	2.30/2.24
Vitamin B12 deficiency	112,542	4,968 (4.4)	3,813 (3.4)	1.0	1.42 (1.36–1.48)	<0.001	2.19/2.06

Each analysis was restricted to patients who had at least one corresponding nutrient measurement during follow-up. Therefore, denominators differ across nutritional deficiencies. Events (%) represent crude proportions of patients with the first laboratory-defined occurrence of each deficiency during the up-to-5-year analytic window and do not account for censoring or competing death. Absolute risk difference was calculated as the event proportion in the COVID-19 group minus that in the control group. Hazard ratios were estimated using Cox proportional hazards models. CI, confidence interval; COVID-19, coronavirus disease 2019; HR, hazard ratio.

### 3.6 Exposure-gradient analysis

In the exposure-gradient analysis, patients with severe COVID-19 consistently demonstrated elevated risks across all outcomes compared with matched controls (Table 8). The magnitude of association was generally greater than that observed in the primary analysis for several outcomes, particularly osteoporotic fracture (HR, 2.13 vs. 1.73 in the primary analysis), femur fracture (HR, 1.85 vs. 1.58), magnesium deficiency (HR, 1.99 vs. 1.77), and mortality (HR, 1.91 vs. 1.59). Risks also remained significantly increased for vitamin D deficiency (HR, 1.77), osteoporosis composite (HR, 1.59), and vitamin B12 deficiency (HR, 1.64) (all *p* < 0.001), although the strength of these associations was similar to that observed in the primary analysis rather than further increased. Mortality was substantially higher among patients with severe COVID-19 (7.48% vs. 4.61%, HR, 1.91, 95% CI, 1.87–1.95).

**Table 8:** Exposure-gradient analysis comparing patients with severe COVID-19 and matched controls.

Outcome	Severe COVID-19 group (*n* = 314,913)	Control group (*n* = 314,913)	HR (95% CI)	*p-*value
	Events (%)	Events (%)		
Vitamin D deficiency	7,887 (2.51)	5,296 (1.68)	1.77 (1.71–1.84)	<0.001
Osteoporosis composite	8,789 (2.79)	6,601 (2.10)	1.59 (1.54–1.64)	<0.001
Osteoporotic fracture	1,522 (0.48)	856 (0.27)	2.13 (1.96–2.32)	<0.001
Femur fracture	3,961 (1.26)	2,500 (0.79)	1.85 (1.76–1.94)	<0.001
Vitamin B12 deficiency	2,669 (0.85)	1,933 (0.61)	1.64 (1.55–1.74)	<0.001
Magnesium deficiency	21,258 (6.75)	12,936 (4.11)	1.99 (1.95–2.04)	<0.001
Any recorded healthcare encounter	263,467 (83.66)	276,163 (87.70)	0.98 (0.97–0.98)	<0.001
Vitamin D measurement^†^	35,297 (11.21)	37,817 (12.01)	1.06 (1.05–1.08)	<0.001
Mortality	23,551 (7.48)	14,531 (4.61)	1.91 (1.87–1.95)	<0.001

Data are presented as number of events (%). Severe COVID-19 was defined as COVID-19 with hospitalization or intensive care unit admission recorded on the index date. Hazard ratios were estimated using Cox proportional hazards models. CI, confidence interval; COVID-19, coronavirus disease 2019; HR, hazard ratio; ICU, intensive care unit. ^†^Event percentages are crude cumulative proportions, whereas hazard ratios incorporate event timing and censoring; therefore, their direction or magnitude may not correspond directly, particularly in the presence of differential mortality and follow-up duration.

### 3.7 Competing-risk analyses

**Figure 2:**
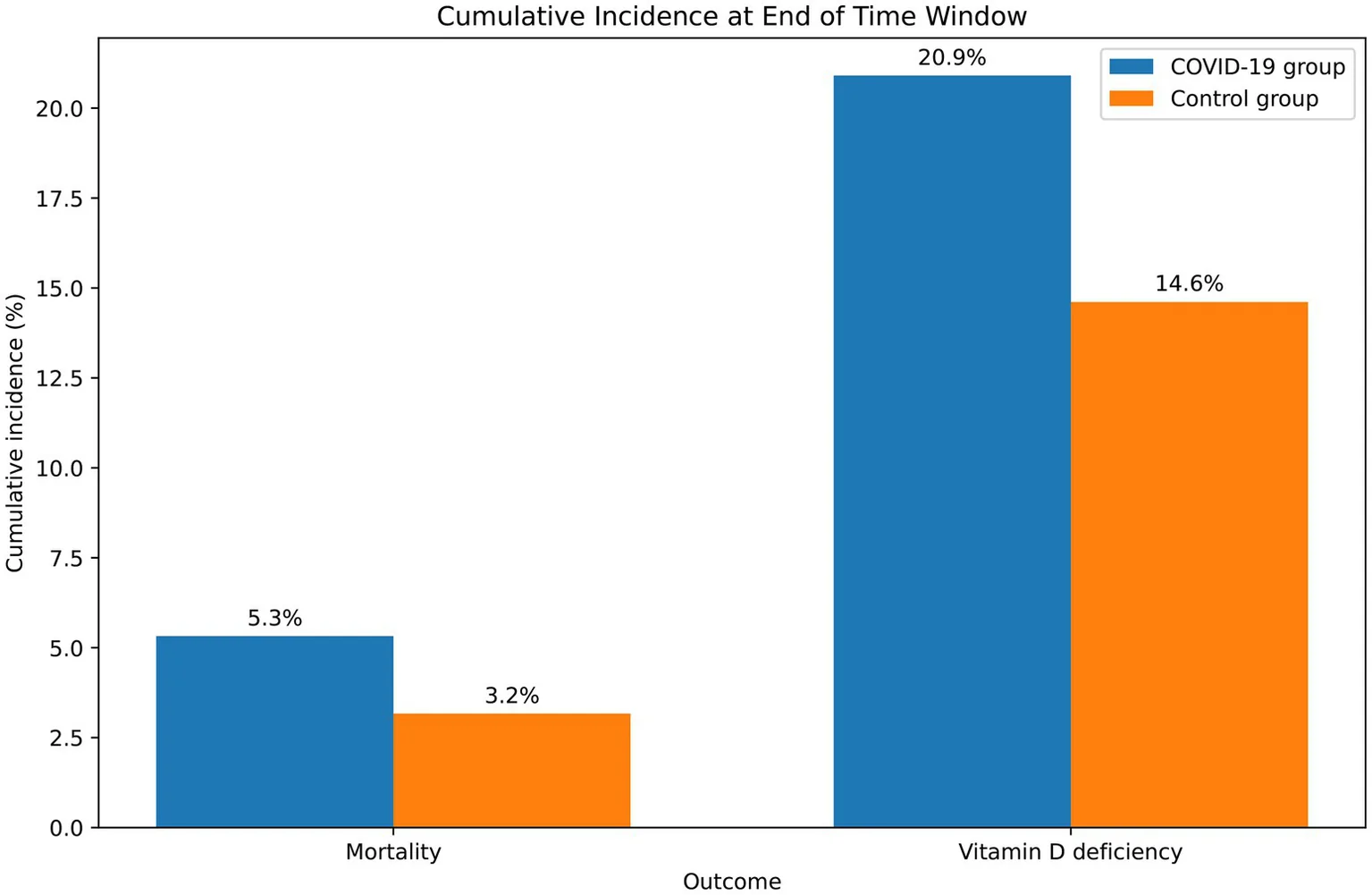
Descriptive competing-risk analysis for vitamin D deficiency with death as a competing event among patients with vitamin D measurement. Bars show Aalen–Johansen cumulative-incidence estimates for vitamin D deficiency and death at the end of the up-to-5-year analytic window. Estimates account for censoring and competing death; full cumulative-incidence curves are not shown.

**Figure 3:**
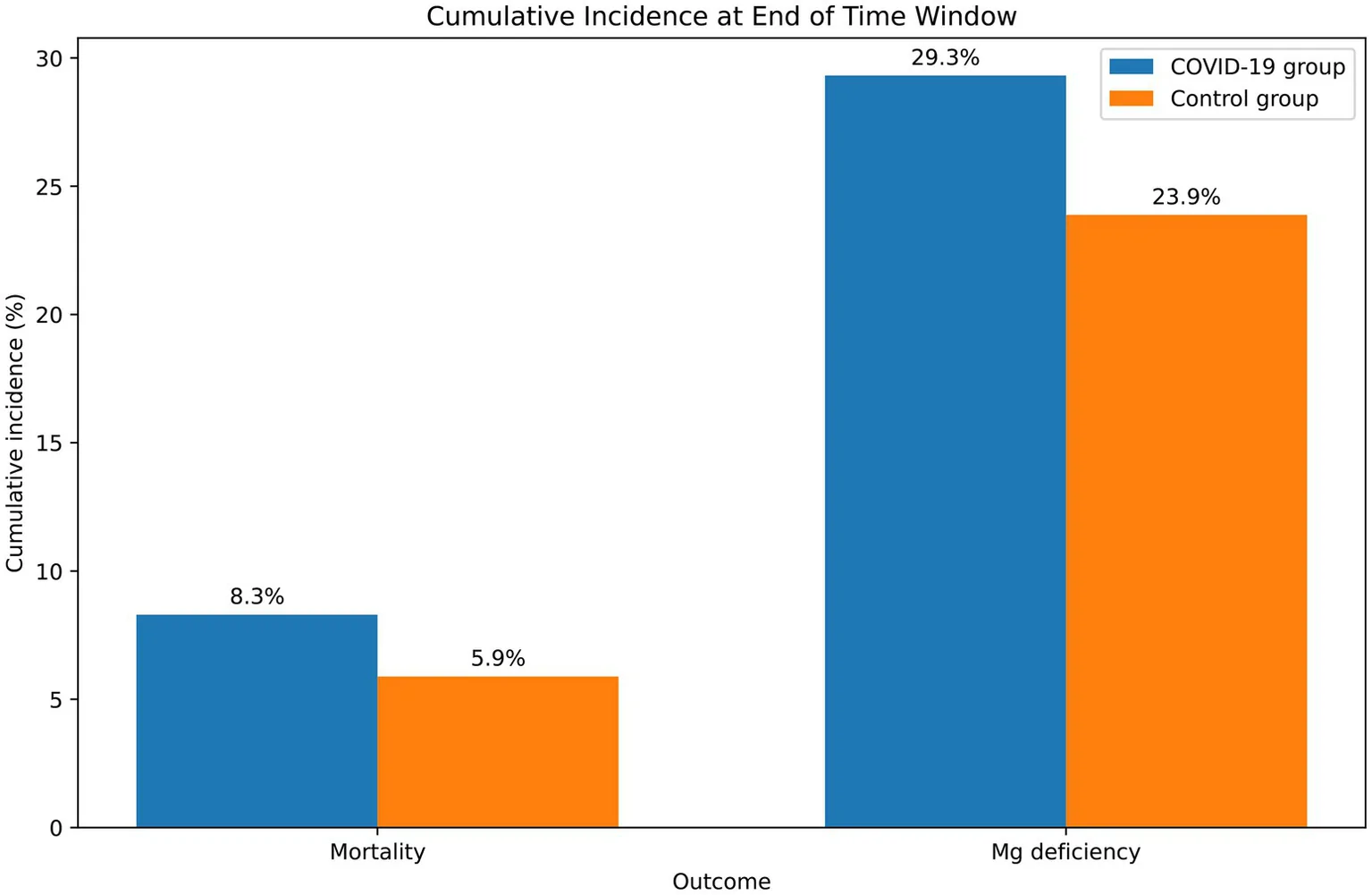
Descriptive competing-risk analysis for magnesium deficiency with death as a competing event among patients with magnesium measurement. Bars show Aalen–Johansen cumulative-incidence estimates for magnesium deficiency and death at the end of the up-to-5-year analytic window. Estimates account for censoring and competing death; full cumulative-incidence curves are not shown.

**Figure 4:**
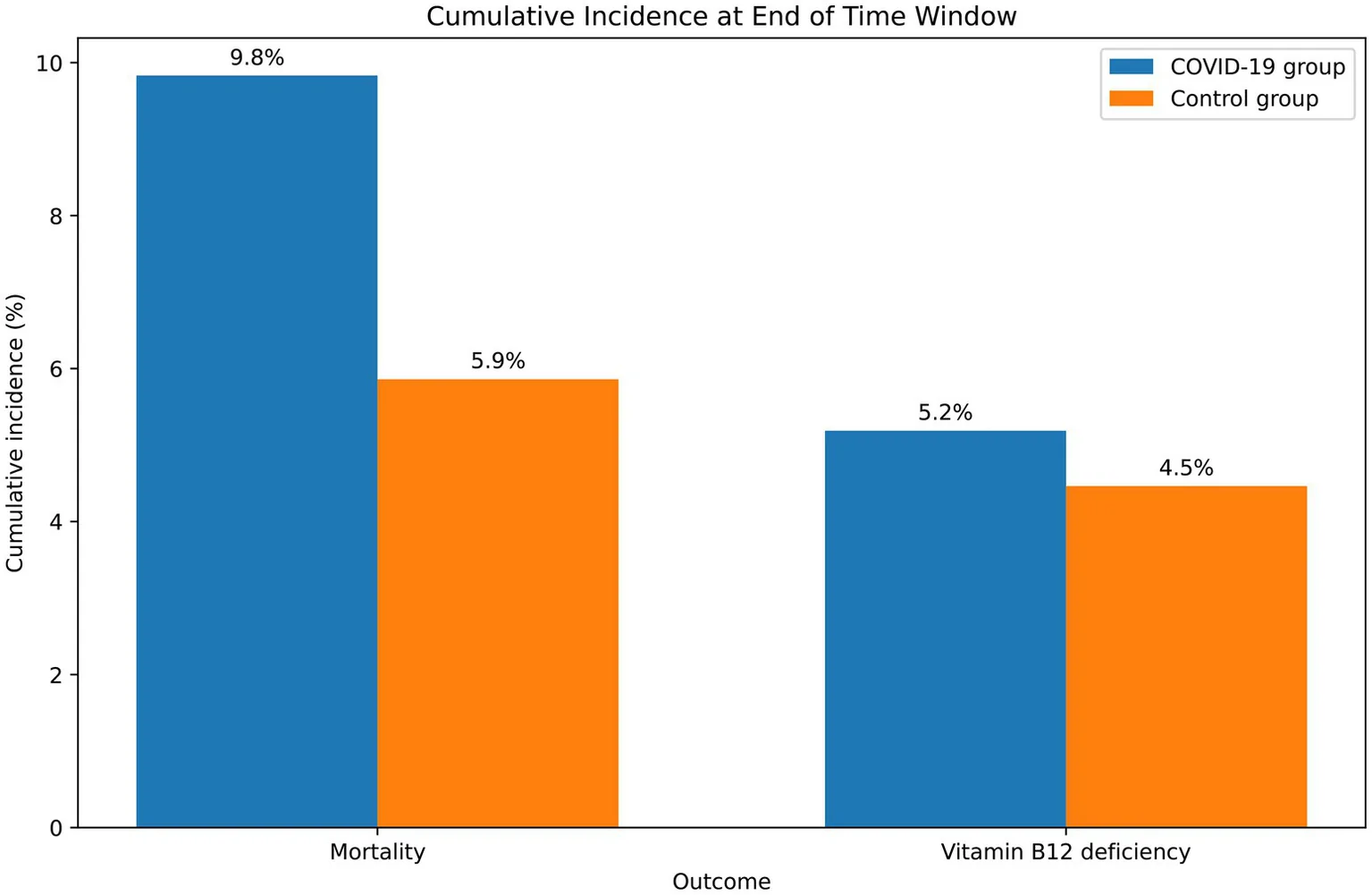
Descriptive competing-risk analysis for vitamin B12 deficiency with death as a competing event among patients with vitamin B12 measurement. Bars show Aalen–Johansen cumulative-incidence estimates for vitamin B12 deficiency and death at the end of the up-to-5-year analytic window. Estimates account for censoring and competing death; full cumulative-incidence curves are not shown.

In descriptive competing-risk analyses restricted to patients with relevant nutrient testing, the cumulative incidence of nutritional deficiency remained higher in the COVID-19 group despite higher competing mortality. For vitamin D deficiency, cumulative incidence was 20.9% in the COVID-19 group and 14.6% in controls, while mortality was 5.3 and 3.2%, respectively. For magnesium deficiency, cumulative incidence was 29.3% versus 23.9%, with mortality of 8.3% versus 5.9%. For vitamin B12 deficiency, cumulative incidence was 5.2% versus 4.5%, with mortality of 9.8% versus 5.9%. These descriptive findings were directionally consistent with the primary Cox analyses but do not constitute a formal between-group competing-risk comparison or eliminate residual competing-risk bias (Figures 2–4).

## 4 Discussion

In this large, multinational cohort of adults with type 2 diabetes, COVID-19 was associated with a higher long-term risk of incident, laboratory-defined micronutrient deficiencies, including vitamin D, magnesium, and vitamin B12 deficiency, as well as a consistent increase in related skeletal outcomes. These associations were observed after a 12-month landmark period, persisted across sensitivity analyses addressing survival and healthcare utilization, and were retained when analyses were restricted to patients who underwent the corresponding nutrient testing during follow-up. Among these tested patients, the absolute risk differences were 6.4, 5.1, and 1.0 percentage points for vitamin D, magnesium, and vitamin B12 deficiency, respectively. Similar patterns observed among patients with severe COVID-19 and in descriptive competing-risk analyses were generally consistent with the primary findings and provide additional context for their interpretation.

Our findings add to the growing literature on COVID-19 and nutritional health by suggesting that micronutrient deficiency should be considered not only as a pre-existing correlate of adverse COVID-19 outcomes, but also as a potential long-term consequence of SARS-CoV-2 infection. Previous studies have primarily investigated vitamin D, magnesium, zinc, or other nutrients in relation to infection susceptibility, hospitalization, intensive care admission, disease severity, and mortality (29–32). Comparatively, relatively little attention has been paid to the development of new-onset micronutrient deficiencies during the post-acute and long-term recovery phases. Existing studies have also tended to focus on general malnutrition, nutritional supplementation strategies, or persistent symptoms associated with post-acute sequelae rather than objectively defined biochemical deficiencies (20, 33, 34). By employing a 12-month landmark design and following participants over an extended period, our study addresses an underexplored dimension of post-COVID care. The observed associations across vitamin D, magnesium, and vitamin B12 deficiency, together with concordant skeletal outcomes, suggest that nutritional disturbances after COVID-19 may involve broader physiological alterations rather than isolated abnormalities in a single nutrient pathway. Nevertheless, these findings should be interpreted cautiously and require confirmation in prospective studies incorporating standardized biochemical assessments and detailed information on dietary intake, supplementation, and lifestyle factors.

The supportive skeletal outcomes observed in this study are consistent with emerging evidence linking COVID-19 to impaired bone health. Prior cohort studies have reported increased risks of new-onset osteoporosis, reduced bone mineral density, and osteoporotic fracture after SARS-CoV-2 infection, particularly among older adults and metabolically vulnerable populations (35–37). Our findings are directionally aligned with these reports (35–37), showing higher risks of osteoporosis, osteoporotic fracture, and femur fracture among patients with type 2 diabetes who survived COVID-19. Importantly, our analysis adds an upstream nutritional perspective by demonstrating parallel increases in vitamin D, magnesium, and vitamin B12 deficiencies, which are biologically relevant to bone metabolism, neuromuscular function, and fracture susceptibility. These concordant skeletal and micronutrient findings strengthen the clinical coherence of the observed associations. However, differences in study populations, follow-up duration, outcome definitions, and ascertainment strategies may explain variation in effect sizes across studies, and the present findings should be interpreted as supportive rather than definitive evidence of a causal bone–nutrition pathway after COVID-19.

Several biological pathways may plausibly link COVID-19 to delayed micronutrient deficiency. Persistent low-grade inflammation and immune activation after SARS-CoV-2 infection may alter micronutrient metabolism, distribution, utilization, and circulating biomarker concentrations, providing a biologically plausible pathway linking COVID-19 to subsequent micronutrient deficiency (38–40). Comparable infection-associated micronutrient disturbances have been reported in other infectious diseases. In treatment-naïve HIV infection, severe vitamin D deficiency was associated with greater inflammatory activity and impaired immune status, whereas a prospective tuberculosis cohort documented frequent deficiencies of vitamin D and several trace elements during the intensive treatment phase (41, 42). These observations support a broader framework in which sustained immune activation, reduced nutritional intake, altered metabolism, and treatment-related effects may contribute to persistent micronutrient abnormalities, although they do not establish a causal parallel with SARS-CoV-2. Gastrointestinal involvement, appetite loss, dysgeusia, altered gut microbiota, and reduced oral intake may also contribute to impaired nutritional intake and absorption, particularly for vitamin B12. Renal tubular dysfunction and metabolic stress may also affect magnesium handling (43, 44), while reduced mobility, limited sunlight exposure, inflammation-related changes in vitamin D metabolism, and interruption of routine supplementation may contribute to vitamin D deficiency. These mechanisms may be especially relevant in patients with type 2 diabetes, who often have chronic inflammation, renal vulnerability, polypharmacy, and baseline metabolic dysregulation (45, 46). However, these mechanisms remain inferential and require confirmation in studies with serial biochemical and physiological measurements.

The stronger associations observed in the post-landmark up-to-3-year analysis followed by attenuation in the 5-year analysis, require cautious interpretation. The proportional-hazards diagnostic for the primary outcome indicated nonproportional hazards, supporting the interpretation that the relative association was not constant over time. Accordingly, the up-to-3-year and up-to-5-year HRs should be viewed as average associations over different analytic windows rather than directly interchangeable estimates of a single constant effect. Several factors may explain this pattern. First, laboratory surveillance after COVID-19 may have increased detection of previously unrecognized or emerging deficiencies, although this is only a partial explanation because overall healthcare visits were not higher in the COVID-19 group. The modestly higher rate of vitamin D measurement in the COVID-19 group (13.31% vs. 12.28%, absolute difference, 1.03 percentage points) nevertheless indicates some degree of differential laboratory ascertainment; however, among patients who underwent vitamin D measurement in the full matched cohorts, the corresponding crude proportions with vitamin D deficiency were 19.5% (16,772/85,853) and 13.5% (10,695/79,237), respectively, closely paralleling the rematched tested-cohort estimates of 19.1 and 12.8%. Second, patients with type 2 diabetes in the control group may gradually develop micronutrient deficiencies because of aging, disease progression, medication exposure, renal dysfunction, or nutritional changes, narrowing the relative difference over time. Third, higher mortality after COVID-19 may selectively remove frailer patients at greatest risk for later deficiency, attenuating long-term estimates. Overall, the attenuation of the association in the up-to-5-year analysis compared with the up-to-3-year analysis likely reflects a combination of biological, clinical, competing-risk, and ascertainment-related factors.

Differential ascertainment remains an important limitation because laboratory-defined deficiencies can only be identified when testing is performed. Patients with COVID-19 may have received more post-infection evaluation, increasing the chance of detecting nutritional abnormalities. However, several findings suggest that differential surveillance alone is unlikely to fully explain the observed associations. First, analyses were restricted to patients who underwent the relevant nutrient testing during follow-up. The associations were attenuated but remained statistically significant for vitamin D, magnesium, and vitamin B12 deficiency. Second, similar results were observed among patients with at least one healthcare visit during follow-up, suggesting that unequal encounter availability alone did not account for the findings. Third, overall healthcare visits were slightly less frequent in the COVID-19 group, whereas nutrient deficiencies remained more common. These observations support the robustness of the association, although they cannot eliminate residual ascertainment bias. Therefore, the findings should be interpreted as evidence of a reproducible post-COVID nutritional signal rather than definitive proof of a causal biological effect.

This study has several strengths. First, the use of a large, multinational federated electronic health record network provided substantial statistical power to evaluate uncommon laboratory-defined deficiencies and skeletal outcomes over extended follow-up. Second, the 12-month landmark design reduced the likelihood that early events reflected pre-existing, undiagnosed deficiency or short-term ascertainment during acute infection. Third, propensity score matching achieved good balance across demographic characteristics, comorbidities, diabetes-related complications, medications, vaccination status, and baseline laboratory markers. Fourth, the study evaluated multiple micronutrients in parallel and incorporated supportive skeletal outcomes, allowing assessment of a broader nutritional and metabolic pattern rather than a single biomarker.

Several limitations should be considered. First, this retrospective electronic health record study is subject to coding errors, incomplete laboratory capture, and heterogeneity in testing practices across healthcare organizations. COVID-19 exposure may also have been misclassified because asymptomatic, home-tested, or externally managed infections were not always captured, particularly early in the pandemic. Nondifferential exposure misclassification would generally be expected to attenuate between-group associations, although the net direction of bias is uncertain because recorded COVID-19 may itself be related to healthcare contact. Because micronutrient deficiencies can only be detected when tests are ordered, the recorded incidence may underestimate the true biological burden and may still be influenced by differential surveillance despite tested-cohort and healthcare-visit sensitivity analyses. These prespecified thresholds define biochemical abnormalities and do not necessarily indicate symptomatic or clinically actionable deficiency. Severity beyond the prespecified cutoffs was not separately stratified. Second, residual confounding cannot be excluded, even after propensity score matching, and the net direction of this bias cannot be determined because the omitted factors may act in opposing directions. Important factors such as dietary intake, nutritional supplementation, sunlight exposure, socioeconomic status, physical activity, corticosteroid exposure, gastrointestinal symptoms, and granular COVID-19 severity markers were not consistently available. In addition, systemic corticosteroids, proton-pump inhibitors or histamine-2 receptor antagonists, and loop or thiazide diuretics were not included in the propensity score model, which may have contributed to residual confounding given their potential effects on bone metabolism, micronutrient absorption, and renal electrolyte handling, particularly in patients with severe COVID-19. Accordingly, the elevated skeletal estimates in the severe-COVID-19 analysis should not be interpreted as evidence of a nutrient-mediated pathway. Third, the landmark design reduced reverse causation and early ascertainment bias but introduced survivorship requirements and may not capture patients with early severe nutritional deterioration or early death. Fourth, the federated network does not allow individual-level chart validation. Generalizability may be limited by the predominantly US-based composition of participating healthcare systems, particularly for Asian and other underrepresented populations. Organization-level geographic distribution and institution types were not available in the aggregate analytic output. Finally, the observational design does not permit causal conclusions, and the secondary, subgroup, tested-cohort, exposure-gradient, and competing-risk analyses should be regarded as exploratory. Future studies incorporating prospectively specified negative-control outcomes may help distinguish biological effects from surveillance-driven associations.

## 5 Conclusion

In this large multinational cohort of patients with type 2 diabetes, COVID-19 was associated with higher long-term risks of incident, laboratory-defined deficiencies of vitamin D, magnesium, and vitamin B12, along with consistently elevated rates of osteoporosis and fracture. These findings suggest that nutritional disturbance may be an under-recognized component of post-acute COVID-19 sequelae in metabolically vulnerable patients. However, residual confounding and differential testing cannot be excluded. Prospective studies with standardized biochemical assessment are needed to confirm causality and determine whether targeted screening or correction improves outcomes.

## Data Availability

The datasets generated and/or analyzed during the current study are not publicly available and are not available from the corresponding author because the data were obtained from the TriNetX Global Collaborative Network under data-use restrictions. TriNetX provides access to de-identified patient-level electronic health record data through a federated research platform, but the underlying patient-level data cannot be exported, deposited in public repositories, or shared by the authors. Access to the data is subject to TriNetX governance, institutional approvals, and applicable privacy and data-use agreements. Requests to access the datasets should be directed to https://live.trinetx.com/.
