# Supplementary material for: COVID-19 and long-term risk of incident laboratory-defined micronutrient deficiency in patients with type 2 diabetes: a multicenter propensity score–matched cohort study
Source: Front Nutr. 2026 Aug 31;13:1915257. doi: 10.3389/fnut.2026.1915257 (PMC13572254; doi:10.3389/fnut.2026.1915257)
Supplement: Supplementary file 1 [file Table_1.DOCX]

**Supplemental Table 1. Codes used to define exclusion criteria**

| **Exclusion criterion** | **Code set / definition** |
| --- | --- |
| Lack of baseline healthcare encounter | Absence of TriNetX visit term (TNX:Visit) during the 5-year baseline period before the index date |
| Type 1 diabetes mellitus before index | ICD-10-CM E10 |
| Gestational diabetes mellitus before index | ICD-10-CM O24.4 |
| Advanced chronic kidney disease before index | ICD-10-CM N18.4, N18.5 |
| End-stage renal disease or dialysis dependence before index | ICD-10-CM N18.6, Z99.2 |
| Bariatric surgery status before index | ICD-10-CM Z98.84 |
| Pre-existing or early vitamin D deficiency | TriNetX laboratory terms TNX:LG25965-1 and TNX:9034; serum 25-hydroxyvitamin D <20 ng/mL, before or within 12 months after index |
| Pre-existing or early magnesium deficiency | LOINC 19123-9; serum magnesium <1.70 mg/dL, before or within 12 months after index |
| Pre-existing or early vitamin B12 deficiency | TriNetX laboratory term TNX:9065; serum vitamin B12 ≤200 pg/mL, before or within 12 months after index |
| Pre-existing or early osteoporosis composite | ICD-10-CM M80, M81, before or within 12 months after index |
| Pre-existing or early osteoporotic fracture | ICD-10-CM M80, before or within 12 months after index |
| Early death during the 12-month landmark period | TriNetX deceased status; ICD-10-CM R99 |
| Evidence of COVID-19 in the control cohort during the study period | ICD-10-CM U07.1 or positive SARS-CoV-2 RNA test (TriNetX laboratory term TNX:9088) between January 1, 2020 and June 30, 2022 |

**Supplemental Table 2. Codes for variables used in propensity score matching**

| **Matching variable** | **Code set / definition** |
| --- | --- |
| Age at index | TriNetX demographic variable |
| Sex | TriNetX demographic variable |
| White | TriNetX race variable; CDC race code 2106-3 |
| Black or African American | TriNetX race variable; CDC race code 2054-5 |
| Asian | TriNetX race variable; CDC race code 2028-9 |
| Body mass index | TriNetX laboratory/vital term TNX:9083; BMI |
| Body mass index >=30 kg/m2 | TriNetX laboratory/vital term TNX:9083; BMI >=30 kg/m2 |
| Essential hypertension | ICD-10-CM I10 |
| Disorders of lipoprotein metabolism and other lipidemias | ICD-10-CM E78 |
| Overweight and obesity | ICD-10-CM E66 |
| Neoplasms | ICD-10-CM C00-D49 |
| Osteoarthritis | ICD-10-CM M15-M19 |
| Ischemic heart diseases | ICD-10-CM I20-I25 |
| Nicotine dependence | ICD-10-CM F17 |
| Diseases of liver | ICD-10-CM K70-K77 |
| Chronic kidney disease | ICD-10-CM N18 |
| Type 2 diabetes mellitus with neurological complications | ICD-10-CM E11.4 |
| Cerebrovascular diseases | ICD-10-CM I60-I69 |
| Type 2 diabetes mellitus with kidney complications | ICD-10-CM E11.2 |
| Heart failure | ICD-10-CM I50 |
| Other chronic obstructive pulmonary disease | ICD-10-CM J44 |
| Type 2 diabetes mellitus with ophthalmic complications | ICD-10-CM E11.3 |
| Type 2 diabetes mellitus with circulatory complications | ICD-10-CM E11.5 |
| Disorders of bone density and structure | ICD-10-CM M80-M85 |
| Alcohol-related disorders | ICD-10-CM F10 |
| Malnutrition | ICD-10-CM E40-E46 |
| Antilipemic agents | TriNetX medication class CV350; ATC C10 |
| Metformin | RxNorm 6809; ATC A10BA |
| Insulins and analogues | ATC A10A |
| SARS-CoV-2 (COVID-19) vaccine | TriNetX medication term 2468231; SARS-CoV-2 mRNA spike-protein vaccine |
| Sodium-glucose cotransporter-2 inhibitors | ATC A10BK |
| Glucagon-like peptide-1 analogues | ATC A10BJ |
| Albumin | TriNetX laboratory term TNX:9045; albumin [mass/volume] in serum, plasma, or blood |
| Albumin >=3.5 g/dL | TriNetX laboratory term TNX:9045; albumin >=3.5 g/dL |
| Estimated glomerular filtration rate | TriNetX laboratory term TNX:8001; creatinine-based eGFR/1.73 m2 |
| Estimated glomerular filtration rate >=60 mL/min/1.73 m2 | TriNetX laboratory term TNX:8001; eGFR >=60 mL/min/1.73 m2 |
| Hemoglobin | TriNetX laboratory term TNX:9014; hemoglobin [mass/volume] in blood |
| Hemoglobin >=12 g/dL | TriNetX laboratory term TNX:9014; hemoglobin >=12 g/dL |
| Hemoglobin A1c | TriNetX laboratory term TNX:9037; hemoglobin A1c/hemoglobin.total in blood |
| Hemoglobin A1c >=9% | TriNetX laboratory term TNX:9037; HbA1c >=9.0% |

Abbreviations: ATC, Anatomical Therapeutic Chemical; BMI, body mass index; CKD, chronic kidney disease; COVID-19, coronavirus disease 2019; eGFR, estimated glomerular filtration rate; HbA1c, hemoglobin A1c; ICD-10-CM, International Classification of Diseases, Tenth Revision, Clinical Modification; LOINC, Logical Observation Identifiers Names and Codes; T2DM, type 2 diabetes mellitus; TNX, TriNetX term.
